# The Psychosocial Burden of Breast Cancer: A Cross-Sectional Study of Associations Between Sleep Quality, Anxiety, and Depression in Turkish Women

**DOI:** 10.3390/jcm14196773

**Published:** 2025-09-25

**Authors:** Ömer Acar, Gamze Goksel, Erol Ozan, Ahmet Anıl Altunbaş, Mustafa Serkan Karakaya, Ferhat Ekinci, Atike Pınar Erdoğan

**Affiliations:** 1 Department of Medical Oncology, Mardin Training and Research Hospital, Vali Ozan Street, 47100 Artuklu, Turkey; 2 Department of Medical Oncology, İzmir Tınaztepe University, 35400 İzmir, Turkey; 3 Department of Psychiatry, Manisa Celal Bayar University, 45030 Manisa, Turkey; 4 Department of Internal Medicine, Manisa Celal Bayar University, 45030 Manisa, Turkeymustafaskarakaya@gmail.com (M.S.K.); 5 Department of Medical Oncology, Manisa Celal Bayar University, 45030 Manisa, Turkey

**Keywords:** breast cancer, sleep quality, anxiety, depression, PSQI, HADS

## Abstract

**Background/Objectives**: Breast cancer remains the most common malignancy among women worldwide, with many patients experiencing persistent psychological symptoms that extend beyond active treatment. Among these, sleep disturbances, anxiety, and depression frequently co-occur and can significantly impair quality of life and treatment adherence. This study aimed to assess the prevalence of poor sleep quality and examine its associations with anxiety and depression in a large cohort of Turkish women with breast cancer. Additionally, the study sought to identify sociodemographic and clinical predictors of sleep disturbance. **Methods**: In this cross-sectional study, 601 women with histologically confirmed breast cancer who were undergoing or had completed active treatment were recruited from a tertiary oncology center in Turkey between January 2023 and December 2023. The mean age of participants was 54 years (range 25–83). More than half of the patients were postmenopausal (56.3%), and 6% had stage IV disease. Sleep quality and psychological distress were assessed using the Pittsburgh Sleep Quality Index (PSQI) and the Hospital Anxiety and Depression Scale (HADS). Descriptive statistics, correlation analyses, and multivariate regression models were employed to identify significant predictors. **Results**: Poor sleep quality (PSQI > 5) was identified in 33.2% of participants. Patients with poor sleep reported significantly higher anxiety and depression scores (*p* < 0.001). Multivariate analysis revealed that being single, having children, undergoing breast-conserving surgery, and elevated anxiety scores were independent predictors of poor sleep. Additionally, marital status, menopausal status, and treatment modality were significantly associated with anxiety and depression scores. **Conclusions**: One-third of Turkish breast cancer patients experience clinically relevant sleep disturbances, which are strongly linked to psychological distress, particularly anxiety. These findings underscore the importance of incorporating routine psychological screening into oncologic care and highlight the need for individualized psychosocial support strategies that aim to improve both emotional well-being and overall clinical outcomes.

## 1. Introduction

Breast cancer is the most commonly diagnosed cancer in women and continues to be the leading cause of cancer-related illness worldwide. According to the GLOBOCAN 2020 data, over 2.3 million new cases of breast cancer were reported, accounting for nearly 12% of all new cancer diagnoses globally [1]. Despite improvements in survival rates due to early detection and advances in treatment such as surgery, chemotherapy, radiotherapy, and targeted therapies, many patients still experience long-term physical and psychological side effects that significantly affect their quality of life [2]. One of the most significant and troubling challenges reported by breast cancer patients is sleep disturbance. These issues are commonly experienced during chemotherapy and radiotherapy, but they often continue into the survivorship phase. Recent studies indicate that 40% to 70% of women with breast cancer experience some form of sleep disruption, which frequently coincides with anxiety and depression [3,4]. Poor sleep quality is not just a subjective issue; it has real clinical consequences. It is associated with immune dysregulation, increased fatigue, cognitive impairment, and lower adherence to treatment [5,6]. These outcomes can adversely affect overall recovery and disease progression. There is also growing evidence of a strong link between sleep disturbances and psychological distress, especially anxiety and depression. These conditions are thought to share common biological pathways, including dysregulation of the hypothalamic–pituitary–adrenal (HPA) axis and increased activity of inflammatory cytokines, as well as behavioral mechanisms like rumination and catastrophizing [7].

Standardized assessment tools, such as the Pittsburgh Sleep Quality Index (PSQI) and the Hospital Anxiety and Depression Scale (HADS), are commonly used to evaluate sleep and psychological symptoms in patients with cancer. The PSQI is a self-reported questionnaire that measures sleep quality and disturbances over one month, while the HADS is a widely used tool for identifying anxiety and depression in hospital outpatient settings. A PSQI score above five indicates that a person is experiencing poor sleep quality. Studies conducted in countries such as China, Morocco, and Iran have consistently found that higher PSQI scores are associated with increased levels of anxiety and depression, highlighting the clinical significance of this symptom cluster [8,9]. Despite the increasing international literature, research on breast cancer patients in Türkiye remains limited. A study conducted by Buyuksimsek et al. found that 78% of women experienced poor sleep quality (PSQI > 5), and more than half showed moderate to severe depressive symptoms. Additionally, a significant positive correlation was identified between sleep quality and both anxiety and depression scores, consistent with global findings [10].

These results indicate that addressing sleep problems, anxiety, and depression is essential for comprehensive cancer treatment. However, in routine clinical settings, these mental health issues are often overlooked in favor of focusing on tumor response and physical symptoms. This neglect can contribute to ongoing psychological distress, reduced quality of life, and potentially poorer clinical outcomes. This study aims to evaluate the prevalence and interrelationship of sleep quality, anxiety, and depression among a large cohort of Turkish women diagnosed with breast cancer. Additionally, it seeks to identify demographic and clinical predictors of psychosocial challenges to inform early interventions and patient-centered supportive care in oncology. The findings from this study may contribute to the development of targeted interventions and personalized care plans, ultimately enhancing the quality of life and clinical outcomes for breast cancer patients.

## 2. Methods

### 2.1. Study Design and Participants

**Figure 1:**
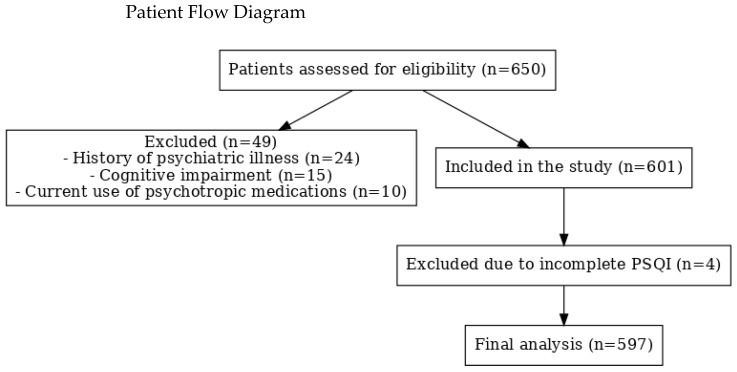
Flowchart of participant selection. Patients assessed for eligibility (n = 650); excluded with reasons (n = 49); included in the study (n = 601); excluded due to incomplete PSQI data (n = 4); final analytic sample (n = 597).

This study was descriptive and cross-sectional, aimed at evaluating the relationship between sleep quality, anxiety, and depression in breast cancer patients in Türkiye. A total of 601 women with histologically confirmed breast cancer, who were either undergoing or had completed active treatment (surgery, chemotherapy, radiotherapy, or hormonal therapy) at a tertiary oncology center between January 2023 and December 2023, were included. After excluding four participants with incomplete PSQI questionnaires, the final analytic sample comprised 597 patients. The sample size was determined during the study period by the available patient population, rather than through an a priori power analysis. Given that 198 patients reported poor sleep quality (PSQI > 5), the events-per-variable (EPV ≥ 10) principle confirmed adequacy for the number of covariates included in multivariable regression analyses. Furthermore, our sample size is larger than or comparable to previously published studies on sleep quality, anxiety, and depression among breast cancer patients, thus providing sufficient statistical power for reliable estimates. The patient selection process is shown in Figure 1. Participants were eligible if they were 18 years or older and capable of understanding and completing the study questionnaires. Those with a history of psychiatric illnesses, cognitive impairments, or current use of psychotropic medications were excluded from the study. All participants provided informed consent before enrollment.

### 2.2. Data Collection

Data were collected through face-to-face interviews using structured questionnaires during routine outpatient visits. The survey included questions about sociodemographic and clinical characteristics, as well as standardized tools to assess sleep quality, anxiety, and depression. On average, completing the questionnaire took approximately 20 to 25 min. All patients had good ECOG performance status (0–1); therefore, ECOG scores were not routinely recorded.

### 2.3. Instruments

#### 2.3.1. Sleep Quality

The quality of sleep was evaluated using the PSQI, a validated tool designed to assess subjective sleep quality over the past month. The PSQI comprises 19 items categorized into seven subdomains: subjective sleep quality, sleep latency, sleep duration, habitual sleep efficiency, sleep disturbances, use of sleep medications, and daytime dysfunction. Each component is scored from 0 to 3, resulting in a global score that ranges from 0 to 21. A total score greater than 5 indicates poor sleep quality. In this study, the Cronbach’s alpha coefficient for internal consistency was 0.84.

#### 2.3.2. Anxiety and Depression

Anxiety and depression were assessed using the Hospital Anxiety and Depression Scale (HADS), which consists of 14 items evenly divided between two subscales: anxiety (HADS-A) and depression (HADS-D). Each item is rated on a 4-point Likert scale (0–3), resulting in subscale scores that range from 0 to 21. The scores are interpreted as follows: 0–7 indicates normal levels, 8–10 indicates borderline (mild) levels, and a score of 11 or higher indicates clinically significant anxiety or depression. In this sample, the Cronbach’s alpha values were 0.85 for the anxiety scale and 0.88 for the depression scale.

#### 2.3.3. Ethical Considerations

This study received approval from the Health Sciences Ethics Committee at Manisa Celal Bayar University (Decision No: E-20478486-050.04.04-452141, Date: 21 December 2022). It was conducted by the principles outlined in the Declaration of Helsinki. Written informed consent was obtained from all participants before data collection. Participation was voluntary, and the confidentiality of the collected data was rigorously maintained.

No AI-assisted technologies were used in the writing or editing of this manuscript.

#### 2.3.4. Statistical Analysis

Statistical analyses were performed using SPSS version 15.0 for Windows (SPSS Inc., Chicago, IL, USA). Descriptive statistics summarized demographic and clinical variables. Continuous variables were assessed for normality using Shapiro–Wilk tests and Q–Q plots; those not normally distributed (e.g., age, BMI) were summarized as medians with interquartile ranges (IQR), while categorical variables were presented as counts and percentages. Group differences in categorical variables were evaluated using the Chi-square test. For continuous variables, the Mann–Whitney U test was applied for two-group comparisons, and the Kruskal–Wallis test for comparisons involving more than two groups. For multi-group comparisons of HADS scores across categorical characteristics, global Kruskal–Wallis tests were followed by pairwise post hoc tests. To control for multiplicity in these post hoc comparisons, significance values were adjusted using the Bonferroni correction, and adjusted *p*-values (adj-p) are reported.

Determinants of poor sleep quality (PSQI > 5) were examined using multivariable logistic regression analysis, with results expressed as odds ratios (ORs) and 95% confidence intervals (CIs). Pairwise correlations among the PSQI global score, HADS-Anxiety (HADS-A), and HADS-Depression (HADS-D) were summarized in a correlation matrix. Depending on the data distribution, Spearman’s ρ was used. Correlation coefficients (r) were reported with corresponding two-sided *p*-values, and statistical significance was defined as α = 0.05.

## 3. Results

The average age of the participants was 54 years, with the majority (79.2%) younger than 65 years. Among them, 89.3% were married, and 80.6% were unemployed. Additionally, 20.8% had a body mass index (BMI) of less than 25 kg/m^2^. Furthermore, 56.3% of the participants were postmenopausal women, and 6% had been diagnosed with stage 4 breast cancer. 29.6% of the patients underwent breast-conserving surgery, 55.4% underwent modified radical mastectomy, and 14.9% had not undergone any surgery for breast cancer. According to the PSQI results, 66.8% of the patients (n = 399) were identified as having good sleep quality, while 33.2% (n = 198) had poor sleep quality. There were no statistically significant differences in demographic characteristics between patients with good and poor sleep quality (Table 1).

A statistically significant difference was found in surgical characteristics: patients with poor sleep quality had a higher rate of breast-conserving surgery, whereas those with good sleep quality had a higher rate of modified radical mastectomy (*p* = 0.029). No significant differences were observed in other disease characteristics (Table 2).

Sleep quality was assessed based on the time since diagnosis when the survey was conducted. No significant differences were found between the groups. However, patients with poor sleep quality had significantly higher scores for anxiety and depression compared to those with good sleep quality (*p* < 0.001 for both). The sleep-related characteristics of both groups are summarized in Table 3. Poor sleep quality was significantly associated with a sleep efficiency of less than 85%, a sleep duration of less than 7 h, a sleep latency of over 30 min, frequent use of sleep medication, greater daytime dysfunction, poor subjective sleep quality, and more frequent sleep disturbances (Table 3).

In the univariate analysis, the risk factors associated with poor sleep quality included undergoing breast-conserving surgery (compared to modified radical mastectomy), receiving adjuvant therapy (compared to no treatment), and having higher scores for anxiety and depression (*p* = 0.008, *p* = 0.032, *p* < 0.001, and *p* < 0.001, respectively). In the multivariate analysis, the following factors were identified as statistically significant risk factors for poor sleep quality: being single (compared to being married), having children, undergoing breast-conserving surgery (compared to modified radical mastectomy), and having higher anxiety scores (*p* = 0.020, *p* = 0.015, *p* = 0.022, and *p* < 0.001, respectively) (see Table 4).

Statistically significant differences in anxiety scores were found based on marital status and treatment type, with *p*-values of 0.049 and 0.015, respectively. Anxiety scores were significantly higher among single patients compared to married patients, as well as among those receiving treatment for metastatic disease compared to patients not undergoing any treatment. Additionally, premenopausal patients showed significantly higher anxiety scores than postmenopausal patients (*p* < 0.001). Moreover, depression scores were significantly higher in patients with a history of receipt of chemotherapy and in those currently receiving chemotherapy, with *p*-values of 0.025 and 0.028, respectively (see Table 5).

Pairwise correlations among sleep quality and psychological outcomes are presented in Table 6. The PSQI global score demonstrated a moderate positive correlation with HADS-Anxiety (r = 0.390, *p* < 0.001) and a weaker but statistically significant positive correlation with HADS-Depression (r = 0.255, *p* < 0.001). Additionally, HADS-Anxiety and HADS-Depression were strongly correlated with each other (r = 0.592, *p* < 0.001).

## 4. Discussion

This study examined the relationships between sleep quality, anxiety, and depression in Turkish breast cancer patients. The results showed that one-third of the participants experienced poor sleep quality. Additionally, individuals with poor sleep reported significantly higher levels of anxiety and depression, which aligns with findings from international studies [3,8].

Our results support previous research showing that sleep problems are a common and distressing issue for breast cancer survivors. Consistent with previous studies, approximately one-third of our sample reported poor sleep quality, as indicated by PSQI scores, a figure comparable to similar research from East Asia and the Middle East [6,9]. Notably, subjective components, such as poor sleep efficiency, sleep duration of less than seven hours, prolonged sleep latency, and daytime dysfunction, were all significantly worse among individuals with poor overall sleep quality. These findings align with studies suggesting that subjective components of sleep (e.g., sleep latency and daytime dysfunction) are the most sensitive to cancer-related distress [11]. An essential finding of this study is the significant correlation between anxiety and poor sleep. Anxiety scores were notably higher in individuals with sleep disturbances, corroborating previous reports that linked sleep issues with psychological vulnerability among cancer patients [7]. In our multivariate analysis, increased anxiety remained an independent predictor of poor sleep, even after adjusting for clinical and sociodemographic variables. This supports the idea that sleep and anxiety may share common neurobiological pathways, such as dysregulation of the hypothalamic–pituitary–adrenal axis and altered cytokine activity [4].

Furthermore, depression scores were also significantly elevated among poor sleepers, as reported in previous research [10]. While anxiety showed a stronger association with poor sleep in our regression model, depression still played a notable role in bivariate comparisons. These results underscore the interconnectedness of sleep, anxiety, and depression in breast cancer patients and support the utility of multidimensional symptom assessment in oncology practice [3]. An interesting finding from our study was the relationship between the type of surgical procedure and sleep quality. Patients who underwent breast-conserving surgery were more likely to report poor sleep quality compared to those who had a modified radical mastectomy. This observation contrasts with some literature suggesting that breast-conserving approaches are associated with a better quality of life [7]. However, it may reflect concerns about body image, fear of recurrence, or inadequate counseling within our cohort. Marital status was identified as a significant predictor of psychological well-being. Single women were more likely to report poor sleep and higher levels of anxiety, which aligns with previous research that emphasizes the protective role of social support in psychological adjustment during cancer survivorship [12]. However, this finding should be interpreted with caution, as the number of single patients in our cohort was limited and the effect size may be unstable. Similarly, individuals currently undergoing chemotherapy or with a history of chemotherapy reported significantly higher levels of depression. This finding supports earlier studies suggesting that cancer treatments can increase psychological distress [13].

The COVID-19 pandemic significantly increased psychological distress among cancer patients, including those with breast cancer. Factors such as social isolation, fear of infection, and disruptions in treatment schedules worsened anxiety and sleep disturbances in this vulnerable group [14,15].

The findings of this study have significant clinical implications. Despite the well-documented negative effects of poor sleep and emotional distress on recovery and treatment adherence, these issues are often underdiagnosed and inadequately addressed in oncology settings [16,17]. Our results highlight the necessity of routine screening for sleep problems, anxiety, and depression using validated tools such as the PSQI and the HADS.

In addition to psychosocial factors, biological elements may also affect breast cancer outcomes. Recent research shows that obesity-related adipokines contribute to tumor growth, and higher chemerin levels in breast cancer cells are linked to increased aggressiveness and poorer prognosis. Combining psychosocial support strategies with biological factors, particularly the roles of obesity and adipokine pathways such as chemerin, could offer a more comprehensive and targeted approach to care [18,19].

This study has several limitations. First, its cross-sectional design precludes any causal inference regarding the relationship between sleep quality, anxiety, and depression. Longitudinal studies are necessary to elucidate the temporal and causal relationships among these variables. Second, the data were collected through self-reported questionnaires, which may be affected by recall bias or social desirability. Third, the study sample was drawn from a single tertiary oncology center in Türkiye, which may limit the generalizability of the findings. Finally, as no multiple comparison correction (e.g., Bonferroni or FDR) was applied, the possibility of Type I error cannot be excluded. Future research should include objective sleep measures (e.g., polysomnography) and recruit more diverse populations to strengthen external validity.

## 5. Conclusions

This study emphasizes the high prevalence of poor sleep quality among Turkish women with breast cancer and its strong association with anxiety and depression. One-third of the participants reported experiencing poor sleep quality, and this was significantly correlated with higher levels of psychological distress. Notably, anxiety was identified as an independent predictor of sleep disturbances, highlighting the need to address emotional well-being as a component of comprehensive cancer care. Identifying and managing sleep and mood disturbances early in the treatment continuum can pave the way for targeted psychosocial interventions that not only alleviate suffering but may also enhance treatment adherence and recovery trajectories. Future longitudinal and interventional studies are warranted to deepen our understanding of these relationships and to evaluate the efficacy of tailored psychosocial support programs in oncology practice.

## Figures and Tables

**Table 1:** Demographic characteristics of patients.

		Total		PSQI Sleep Quality				
				≤5 Good n = 399 (%66.8)		>5 Bad n = 198 (%33.2)		*p*
Age Median (Min–Max)		54 (25–83)		54 (25–83)		55 (34–82)		0.464
BMI Median (Min–Max)		28.9 (16.2–49.5)		28.7 (16.2–49.5)		29.6 (18.1–49.5)		0.308
		n	%	n	%	n	%	*p*
Age	<65	473	79.2	320	80.2	153	77.3	0.406
	≥65	124	20.8	79	19.8	45	22.7	
BMI	<25	124	20.8	84	21.1	40	20.2	0.809
	≥25	473	79.2	315	78.9	158	79.8	
Marital status	Married	527	89.3	353	89.6	174	88.8	0.255
	Single	13	2.2	6	1.5	7	3.6	
	Widow	50	8.5	35	8.9	15	7.7	
Child status	No	27	4.8	19	5.1	8	4.2	0.615
	Yes	536	95.2	352	94.9	184	95.8	
Job	Housewife	470	80.6	310	80.3	160	81.2	0.691
	Civil Servant	29	5.0	21	5.4	8	4.1	
	Worker	55	9.4	38	9.8	17	8.6	
	Retired	29	5.0	17	4.4	12	6.1	
Chronic disease	No	332	56.2	230	58.2	102	52.0	0.154
	Yes	259	43.8	165	41.8	94	48.0	
Menopause status	Peri-Menopause	261	43.7	171	42.9	90	45.5	0.547
	Post-Menopause	336	56.3	228	57.1	108	54.5	
Smoking	Never	447	77.3	299	77.5	148	77.1	0.944
	Drinks	99	17.1	65	16.8	34	17.7	
	Quit	32	5.5	22	5.7	10	5.2	

BMI: Body mass index, PSQI: Pittsburgh Sleep Quality Index.

**Table 2:** Clinical characteristics of patients.

		Total		PSQI Sleep Quality				
				≤5 Good n = 399 (%66.8)		>5 Bad n = 198 (%33.2)		*p*
Clinical stage	Stage 1	117	19.6	72	18.0	45	22.7	0.595
	Stage 2	321	53.8	219	54.9	102	51.5	
	Stage 3	123	20.6	83	20.8	40	20.2	
	Stage 4	36	6.0	25	6.3	11	5.6	
Surgery	No	89	14.9	59	14.8	30	15.2	**0.029**
	BCS	177	29.6	105	26.3	72	36.4	
	MRM	331	55.4	235	58.9	96	48.5	
Neoadjuvant therapy	No	429	71.9	283	70.9	146	73.7	0.472
	Yes	168	28.1	116	29.1	52	26.3	
Tumor localization	Right	296	49.6	193	48.4	103	52.0	0.497
	Left	292	48.9	201	50.4	91	46.0	
	Bilateral	9	1.5	5	1.3	4	2.0	
Treatment	No treatment	94	15.7	72	18.0	22	11.1	0.182
	Adjuvant	364	61.0	236	59.1	128	64.6	
	Neoadjuvant	59	9.9	38	9.5	21	10.6	
	Metastatic	80	13.4	53	13.3	27	13.6	
Chemotherapy	No	57	9.5	42	10.5	15	7.6	0.248
	Yes	540	90.5	357	89.5	183	92.4	
Radiotherapy	No	228	38.2	157	39.3	71	35.9	0.409
	Yes	369	61.8	242	60.7	127	64.1	

PSQI: Pittsburgh Sleep Quality Index, BCS: Breast-conserving surgery, MRM: modified radical mastectomy. Values in bold indicate statistically significant differences at *p* < 0.05.

**Table 3:** Correlation between the PSQI (components and total score) and the HADS components.

		Total		PSQI Sleep Quality				
				≤5 Good n = 399 (%66.8)		>5 Bad n = 198 (%33.2)		*p*
**PSQI sleep quality** Median (Min–Max)		4 (0–19)		3 (0–5)		9 (6–19)		<0.001
**Anxiety score** Median (Min–Max)		6 (0–21)		5 (0–19)		9 (0–21)		<0.001
**Depression score** Median (Min–Max)		5 (0–21)		4 (0–18)		7 (0–21)		<0.001
		n	%	n	%	N	%	*p*
**Diagnosis time**	≤2 years	345	57.8	230	57.6	115	58.1	0.919
	>2 years	252	42.2	169	42.4	83	41.9	
**Habitual sleep efficiency**	≥85%	496	83.1	392	98.2	104	52.5	<0.001
	75% to 84%	33	5.5	5	1.3	28	14.1	
	65% to 74%	24	4.0	2	0.5	22	11.1	
	≤64%	44	7.4	0	0.0	44	22.2	
Sleep duration	>7 h	432	72.4	357	89.5	75	37.9	<0.001
	6.0–6.9 h	78	13.1	35	8.8	43	21.7	
	5.0–5.9 h	50	8.4	5	1.3	45	22.7	
	<5 h	37	6.2	2	0.5	35	17.7	
Sleep latency	<15 min	249	41.7	216	54.1	33	16.7	<0.001
	16–30 min	185	31.0	135	33.8	50	25.3	
	31–60 min	96	16.1	41	10.3	55	27.8	
	>60 min	67	11.2	7	1.8	60	30.3	
Sleep medication use	0	523	87.6	382	95.7	141	71.2	<0.001
	1	20	3.4	9	2.3	11	5.6	
	2	11	1.8	4	1.0	7	3.5	
	3	43	7.2	4	1.0	39	19.7	
Daytime dysfunction	0	491	82.2	368	92.2	123	62.1	<0.001
	1	54	9.0	24	6.0	30	15.2	
	2	33	5.5	6	1.5	27	13.6	
	3	19	3.2	1	0.3	18	9.1	
Subjective sleep quality	0	177	29.6	162	40.6	15	7.6	<0.001
	1	284	47.6	215	53.9	69	34.8	
	2	103	17.3	21	5.3	82	41.4	
	3	33	5.5	1	0.3	32	16.2	
Sleep disturbance	0	11	1.8	11	2.8	0	0.0	<0.001
	1	249	41.7	223	55.9	26	13.1	
	2	264	44.2	158	39.6	106	53.5	
	3	73	12.2	7	1.8	66	33.3	

PSQI: Pittsburgh Sleep Quality Index, HADS: Hospital Anxiety and Depression Scale.

**Table 4:** Univariate and Multivariate Logistic Regression Analyses of Factors Associated with Poor Sleep Quality.

		**Univariate**			**Multivariate**		
		***p***	**OR**	**95% C.I**	***p***	**OR**	**95% C.I**
**Age**		0.438	1.006	0.991–1.021	0.214	1.020	0.988–1.053
**BMI**		0.307	1.017	0.985–1.050	0.609	1.011	0.970–1.054
**Marital status** (Ref: Married)		0.274			0.034		
	Single	0.127	2.367	0.784–7.149	0.020	13.29	1.494–118.26
	Widow	0.664	0.869	0.462–1.635	0.242	0.628	0.289–1.368
**Having children** (Ref: No)	Yes	0.616	1.241	0.533–2.890	0.015	8.499	1.505–47.99
**Occupation** (Ref: Housewife)		0.693			0.711		
	Civil servant	0.477	0.738	0.320–1.703	0.887	1.074	0.399–2.891
	Worker	0.642	0.867	0.474–1.584	0.738	0.879	0.414–1.869
	Retired	0.421	1.368	0.638–2.934	0.268	1.721	0.659–4.495
Chronic disease (Ref: No)	Yes	0.154	1.285	0.910–1.813	0.539	1.153	0.732–1.817
Menopausal status (Ref: Peri-)	Post-	0.547	0.900	0.639–1.268	0.279	0.688	0.350–1.353
Smoking (Ref: Non-smoker)		0.944			0.585		
	Smoker	0.814	1.057	0.668–1.673	0.440	0.798	0.450–1.415
	Quit	0.829	0.918	0.424–1.989	0.436	0.695	0.278–1.735
Stage at survey (Ref: Stage 1)		0.596			0.984		
	Stage 2	0.191	0.745	0.480–1.158	0.885	0.957	0.526–1.740
	Stage 3	0.337	0.771	0.454–1.310	0.867	1.067	0.501–2.271
	Stage 4	0.390	0.704	0.316–1.568	0.977	1.023	0.212–4.947
Surgery (Ref: MRM)		0.029			0.056		
	None	0.390	1.245	0.755–2.051	0.526	1.661	0.347–7.960
	BCS	0.008	1.679	1.145–2.461	0.022	1.887	1.098–3.243
Treatment (Ref: No treatment)		0.189			0.245		
	Adjuvant	0.032	1.775	1.051–2.997	0.068	1.853	0.956–3.592
	Neoadjuvant	0.105	1.809	0.884–3.699	0.824	1.232	0.195–7.775
	Metastatic	0.132	1.667	0.857–3.243	0.881	1.083	0.382–3.067
History of chemotherapy (Ref: No)	Yes	0.250	1.435	0.775–2.657	0.559	1.276	0.564–2.886
History of radiotherapy (Ref: No)	Yes	0.409	1.160	0.815–1.652	0.820	0.934	0.517–1.687
Currently receiving chemotherapy (Ref: No)	Yes	0.287	1.220	0.846–1.759	0.370	1.347	0.702–2.583
Anxiety score		<0.001	1.235	1.177–1.295	<0.001	1.216	1.141–1.295
Depression score		<0.001	1.165	1.114–1.218	0.093	1.057	0.991–1.127
Time from diagnosis to survey		0.182	1.035	0.984–1.088	0.353	1.036	0.962–1.116

BMI: Body mass index, BCS: Breast-conserving surgery, MRM: modified radical mastectomy, OR: odds ratio.

**Table 5:** Comparison of Anxiety and Depression Scores According to Sociodemographic and Clinical Variables.

		Anxiety Score		Depression Score	
		Median (Min–Max)	*p*	Median (Min–Max)	*p*
Age	<65 years	6 (0–21)	0.182	5 (0–21)	0.715
	≥65 years	6 (0–18)		5 (0–20)	
Marital status	Married	6 (0–21)	0.049	5 (0–21)	0.144
	Single	9 (4–16) ^a^		5 (1–14)	
	Widow	6 (0–21)		6 (0–19)	
Having children	No	7 (0–17)	0.094	4 (0–16)	0.929
	Yes	6 (0–21)		5 (0–21)	
Occupation	Housewife	6 (0–21)	0.779	5 (0–21)	0.678
	Civil servant	7 (0–15)		5 (0–13)	
	Worker	6 (0–18)		5 (0–20)	
	Retired	6 (1–17)		4 (0–20)	
BMI	<25	6 (0–21)	0.084	4 (0–21)	0.298
	≥25	6 (0–21)		5 (0–20)	
Chronic disease	No	6 (0–21)	0.882	5 (0–21)	0.565
	Yes	6 (0–21)		5 (0–20)	
Menopausal status	Pre-	7 (0–21)	<0.001	5 (0–21)	0.808
	Post-	6 (0–21)		5 (0–20)	
Smoking	Non-smoker	6 (0–21)	0.086	5 (0–20)	0.895
	Smoker	7 (0–21)		4 (0–21)	
	Quit	7 (1–18)		6 (0–16)	
Clinik stage	Stage 1	6 (0–19)	0.636	5 (0–20)	0.231
	Stage 2	6 (0–21)		5 (0–21)	
	Stage 3	6 (0–19)		5 (0–17)	
	Stage 4	6 (2–18)		7 (0–20)	
Surgery	None	6 (0–21)	0.869	6 (0–21)	0.330
	BCS	6 (0–19)		5 (0–20)	
	MRM	6 (0–21)		5 (0–20)	
Treatment	No treatment	5 (0–18)	0.015	5 (0–20)	0.150
	Adjuvant	6 (0–21)		5 (0–20)	
	Neoadjuvant	6 (0–21)		6 (0–1)	
	Metastatic	6 (1–21) ^b^		6 (0–20)	
History of chemotherapy	No	6 (1–18)	0.456	4 (0–14)	0.025
	Yes	6 (0–21)		5 (0–21)	
History of radiotherapy	No	6 (0–21)	0.439	5 (0–21)	0.492
	Yes	6 (0–21)		5 (0–20)	
Currently receiving chemotherapy	No	6 (0–19)	0.252	5 (0–20)	0.028
	Yes	6 (0–21)		6 (0–21)	
Diagnosis to survey time	≤2 years	6 (0–21)	0.941	5 (0–21)	0.775
	>2 years	6 (0–21)		5 (0–20)	

^a^ Significantly different from the married group (*p* = 0.015) ^b^ Significantly different from the no treatment group (*p* = 0.001). *p*-values for pairwise comparisons were adjusted using the Bonferroni correction following significant Kruskal–Wallis tests.

**Table 6:** Correlation matrix (r and *p*-values).

	PSQI Global Score	HADS-Anxiety (HADS-A)	HADS-Depression (HADS-D)
PSQI Global Score		r = 0.390, *p* < 0.001	r = 0.255, *p* < 0.001
HADS-Anxiety (HADS-A)	r = 0.390, *p* < 0.001		r = 0.592, *p* < 0.001
HADS-Depression (HADS-D)	r = 0.255, *p* < 0.001	r = 0.592, *p* < 0.001	

Note: Correlations are based on Spearman’s ρ; all *p*-values are two-sided.

## Data Availability

The datasets generated and/or analyzed during the current study are available from the corresponding author upon reasonable request.
